# Focal Hepatic Hypoperfusion After Normothermic Machine Perfusion of Liver Grafts Is Associated with a Higher Comprehensive Complication Index

**DOI:** 10.3390/bioengineering13070729

**Published:** 2026-06-24

**Authors:** Felicia Kneifel, Felix Becker, Qing Wen Lin, Carsten Szardenings, Sebastian Kubasch, Arne Riegel, Haluk Morgül, Isabelle Flammang, Shadi Katou, Andreas Pascher, Philipp Houben

**Affiliations:** 1 Department of General, Visceral and Transplant Surgery, University Hospital Münster, 48149 Münster, Germany; 2 Transplant Organ Recovery Center (TORC), University Hospital Münster, 48149 Münster, Germany; 3 Institute of Biostatistics and Clinical Research, University Münster, 48149 Münster, Germany; 4 Clinic for Radiology, University Hospital Münster, 48149 Münster, Germany

**Keywords:** focal hepatic hypoperfusion, liver transplantation, machine perfusion

## Abstract

**Background:** Normothermic machine perfusion (NMP) is increasingly being used to improve organ utilization in liver transplantation (LT). However, its non-physiological perfusion setting may cause focal hepatic hypoperfusion (FHH), which remains insufficiently characterized in terms of its incidence, risk factors, and clinical impact. **Methods:** Data on liver grafts that underwent NMP prior to LT at the Department of General, Visceral, and Transplant Surgery, University Hospital Münster, between October 2019 and August 2024 were retrospectively analyzed. Recipients who underwent contrast-enhanced computed tomography within 30 days post-LT were included. The primary outcomes were the Comprehensive Complication Index (CCI) and overall graft survival rate. Ninety-one patients met the inclusion criteria and were stratified according to the presence of FHH in the FHH+ (n = 27) and FHH- (n = 64) groups. **Results:** FHH was detected in 29.7% of the grafts. Higher graft weight was the only independent predictor of FHH. In addition, graft weight correlated with the extent of FHH (τ = 0.40, *p* < 0.001). FHH did not affect graft or patient survival but was associated with higher CCI scores (*p* = 0.001) and prolonged intensive care unit length of stay (*p* = 0.028). **Conclusions:** FHH is a common radiological finding after NMP. Although it does not affect graft loss, its association with a higher complication burden warrants further attention. Whether avoiding NMP in very heavy grafts could reduce the incidence of FHH remains to be determined.

## 1. Introduction

Focal hepatic hypoperfusion (FHH) is a radiological finding after liver transplantation (LT) that, unlike hepatocyte necrosis, does not necessarily indicate irreversible damage. While necrosis is diagnosed microscopically as an adverse histopathological feature [1], hypoperfusion appears on computed tomography (CT) scans as localized, hypodense, and non-enhancing areas. These ischemic regions may progress to necrosis; however, FHH can also be a transient and benign phenomenon that resolves over time [2,3].

Over the past decade, normothermic machine perfusion (NMP) has gained global acceptance as an advanced modality for liver grafts [4,5,6]. Despite these advantages, NMP presents several challenges. During perfusion, the graft was positioned on the anterior and cranial surfaces, within a plastic cradle. Limited connective tissue support in this configuration predisposes to compression of portal venous structures, resulting in heterogeneous perfusion. Furthermore, reliance on gravity alone is inadequate to sustain the physiological pressure gradient between the portal and hepatic veins [7], leading to diminished hepatic blood flow and formation of abnormal intraparenchymal pressure zones [8,9].

Historically, Velasques et al. demonstrated that the application of external pressure to the liver improves perfusion quality [10]. Subsequently, Neuhaus et al. refined this approach by implementing sinusoidal pressure waveforms to mimic the intra-abdominal environment, thereby facilitating homogeneous perfusion through complete venous drainage [7,10]. More recently, Eshmuminov et al. demonstrated in a seven-day long-term NMP pilot study of discarded human livers that diaphragm-simulating oscillations of the organ chamber prevented FHH [11]. Despite these advancements, these strategies have not yet been incorporated into commercially available machine perfusion systems.

Recent findings indicate that compression of the liver parenchyma due to nonanatomic positioning during perfusion induces characteristic post-transplant changes, including FHH [2,3,12,13,14]. The ‘cradle sign’, first described by Richards et al. in 2021 [12], refers to compression marks on the liver caused by its placement on the anterior and cranial surfaces within a plastic cradle during machine perfusion. Richards et al. reported an incidence of <5% in perfused livers at their center. Despite their appearance on CT scans, these livers were successfully transplanted when the other viability criteria were met [12].

In contrast, Martin et al. identified a related incidental radiological phenomenon of FHH, termed ‘cradle compression’ (CC), characterized by heterogeneous opacification on contrast-enhanced CT within 14 days post-transplantation, predominantly affecting liver segments VII and VIII [2]. Although various hypotheses have been proposed [2,3,13,14], the exact pathophysiology of FHH remains unknown.

This study aimed to investigate the incidence, risk factors, related complications, and short- and long-term outcomes of FHH after NMP of liver grafts, without evidence of macrovascular complications.

## 2. Materials and Methods

### 2.1. Study Population and Study Design

This retrospective analysis included liver transplants that underwent NMP before LT at the Department of General, Visceral, and Transplant Surgery, University Hospital Münster, Germany, between October 2019 and August 2024.

NMP was performed using a back-to-base approach with OrganOx Metra as previously described [15,16]. Our institutional protocol required a minimum of 6 h of NMP. Organ viability was assessed according to the Münster Viability Protocol, using a perfusate lactate concentration of ≤2.0 mmol/L after 6 h of NMP as the criterion for graft acceptance [15]. All the patients received standard postoperative care.

The inclusion criterion was a biphasic CT scan of the upper abdomen within 30 days post-LT. Exclusion criteria included retransplantation (reLT) and vascular complications, such as hepatic artery thrombosis, arterial dissection, or portal vein thrombosis.

This study was conducted in accordance with the ethical principles of the Declaration of Helsinki. This study was approved by the local Ethics Committee (Ethics Committee of the Medical Association of Westphalia-Lippe and the Westphalian Wilhelms University, No. 2019-673-f-S).

### 2.2. Demographics

All liver grafts were procured from heart-beating, brain-dead donors (DBD). Donor characteristics, including age, sex, and body mass index (BMI), were obtained from the Eurotransplant Network Information System (ENIS), and the Eurotransplant Donor Risk Index (ET-DRI) was calculated accordingly [17].

### 2.3. Liver Weight

Liver weight, recorded after back-table preparation and prior to cannulation, was retrospectively retrieved from the surgical reports. For eight allografts, liver weight data were unavailable and were therefore estimated based on the donor’s body surface area (BSA), calculated using the Mosteller formula: BSA (m^2^): √([weight (kg) × height (cm)]/3600) [18]. The estimated liver weight was calculated using the following equation: Liver weight (g) = 772 × BSA [19].

### 2.4. Radiological Assessment of Focal Hepatic Hypoperfusion

**Figure 1:**
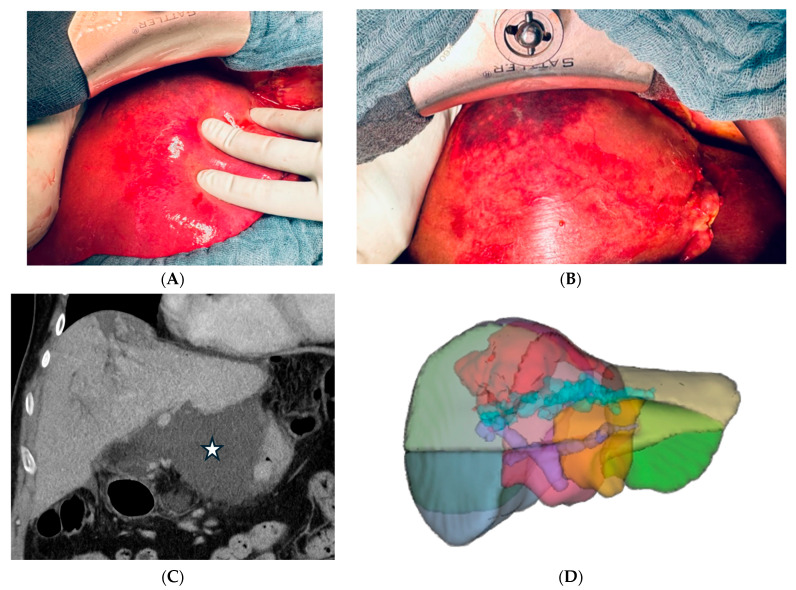
Focal hepatic hypoperfusion (FHH). (**A**,**B**) Intraoperative images: (**A**) FHH involving liver segments VII and VIII, 90 min post-reperfusion, and (**B**) on postoperative day 2. (**C**) Contrast-enhanced coronal CT (postoperative day 2) showing a well-defined hypodense area corresponding to FHH. (**D**) Three-dimensional reconstruction illustrating Couinaud liver segmentation with color-coded segments. FHH (red) is localized in segments IVa, VII, and VIII. All images depict the same liver graft. On postoperative day two, CT imaging and subsequent reoperation were performed due to a perihepatic hematoma (white star). FHH: focal hepatic hypoperfusion.

FHH was identified based on heterogeneous opacification patterns on contrast-enhanced CT scans of grafts subjected to NMP (Figure 1).

CT scans performed within 30 days after LT were included in the study. Radiological perfusion abnormalities range from isolated linear hypoattenuations to diffuse parenchymal contusions. Volumetric assessments of the entire liver graft and the corresponding FHH areas (Figure 1) were independently performed by a radiologist with five years of experience in abdominal imaging and a board-certified senior consultant in radiology, who also validated the measurements.

### 2.5. Outcome Measures

The primary outcome measures included postoperative complications, assessed using the Comprehensive Complication Index (CCI) [20], and overall graft survival. According to a standardized institutional protocol, the CCI was prospectively recorded at hospital discharge for all patients undergoing LT.

Secondary endpoints included overall patient survival, primary nonfunction (PNF), early allograft dysfunction (EAD) as defined by Olthoff et al. [21], reLT rates within the first year, postoperative infections, peak postoperative Aspartate Aminotransferase (AST) and Alanine Aminotransferase (ALT) levels, intensive care unit (ICU) length of stay, and total hospital length of stay.

### 2.6. Statistical Analysis

All analyses were performed using R software (version 4.4.1; R Foundation for Statistical Computing, Vienna, Austria) and GraphPad Prism (version 9.3.1; GraphPad Software, San Diego, CA, USA). Missing data were minimal and were addressed through a complete case analysis.

Nonparametric methods were applied, including Fisher’s exact test, Wilcoxon–Mann–Whitney test, Kaplan–Meier estimator, log-rank test, and Kendall’s tau. The t-test was used for group comparisons of continuous variables where appropriate. The local significance level was set at α = 0.05, and the confidence intervals (CI) were reported at the 95% level.

We aimed to define a liver weight cutoff to enable risk stratification by identifying the threshold that best discriminates grafts with an increased risk of FHH from those without. The cut-off was determined by maximizing Youden’s J [22] to achieve optimal discrimination through a balanced consideration of sensitivity and specificity.

To account for potential confounding factors, such as cold ischemia time (CIT), Model for End-Stage Liver Disease (MELD) score, donor cardiopulmonary resuscitation (CPR), donor and recipient sex, age, and body mass index (BMI), multivariable regression models were used whenever the number of events was sufficient, and model assumptions were adequately met.

Predictors for each outcome were determined using backward elimination, which was terminated when the likelihood ratio test yielded *p* < 0.15 for all remaining variables in the generalized models, or when the adjusted R^2^ decreased in the linear models. Details specific to each outcome are provided in the Results section.

## 3. Results

Between October 2019 and August 2024, 166 liver allografts underwent NMP, of which 149 were subsequently transplanted. The average NMP duration was 14.4 ± 4.9 h. Postoperatively, 115 recipients (77.2%) underwent biphasic CT of the upper abdomen within 30 days after LT. Twenty-four patients were excluded based on the following predefined criteria: reLT (n = 14) or vascular complications (n = 10). After applying all inclusion and exclusion criteria, 91 patients were eligible for the analysis. To assess the potential selection bias associated with clinically indicated postoperative CT imaging, a comparative analysis of patients who did and did not undergo CT imaging within 30 days after LT was performed. The results of this analysis are provided in the Supplementary Material (Table S1).

FHH was identified in 27 patients (29.7%), most commonly involving liver segments VII and VIII (21 patients, 77.8%). Patients were stratified into two groups according to the presence of FHH: (1) the FHH+ group (FHH+, n = 27) and (2) the FHH- group (FHH-, n = 64). The median time from LT to CT imaging was 6 days (IQR, 4–15) in the FHH+ group and 9 days (IQR, 5–12) in the FHH− group, with no significant difference between groups (*p* = 0.439). Among the eight grafts with estimated rather than directly measured liver weight, seven were assigned to the FHH− group and one to the FHH+ group.

The median total liver volume was 2360 mL (IQR, 1899–2869 mL), with a median FHH volume of 82.8 mL (IQR, 11.4–391.2), corresponding to a median FHH proportion of 4.4% (IQR, 0.66–10.9).

### 3.1. Study Population Characteristics

The donor and recipient characteristics were comparable between the FHH+ and FHH groups. Notably, both donor and recipient BMI were marginally higher in the FHH+ group than in the FHH- group, with only donor BMI reaching statistical significance. The mean graft weight was significantly higher in the FHH+ group than in the FHH- group (1847 vs. 1634 g, *p* = 0.043) (Table 1).

### 3.2. Factors Affecting Focal Hepatic Hypoperfusion

To assess FHH, multivariable logistic regression models were fitted for all LT included in the study. Backward elimination removed all predictors except graft weight. The effect of graft weight was modeled per 100 g, with each additional 100 g associated with a 14% increase in odds ratio (OR 1.14; 95% CI 1.02–1.29; *p* = 0.026) (Table 2).

The univariate model demonstrated a modest predictive performance, with an AUC of 0.65. Youden’s J-optimized cutoff [22] for liver weight was 1958 g, yielding low sensitivity (0.48), but high specificity (0.86). No generalized multivariable regression model provided an adequate fit for the volumetric proportion of FHH in all included livers. Hence, we analyzed the rank correlation between the percentage and predictors as non-parametric univariable equivalents. Higher graft weight (τ = 0.40, *p* < 0.001) was significantly associated with a higher percentage of FHH. All considered predictors are listed in Table 2.

**Figure 2:**
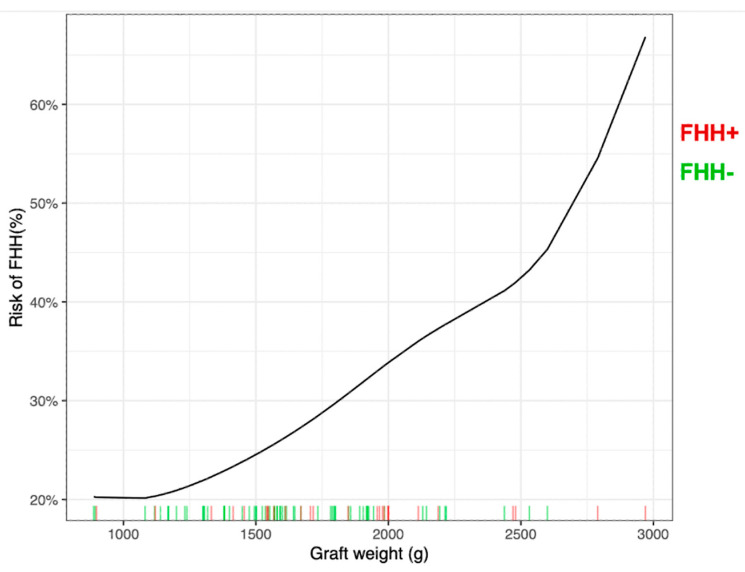
Estimated risk of FHH by graft weight. Risk was calculated as the moving weighted average of the FHH indicator variable (1 = FHH, 0 = no FHH) using a Gaussian kernel with a standard deviation of 300 g. The x-axis shows the observed graft weight; grafts without FHH are shown in green; grafts with FHH are shown in red. FHH: focal hepatic hypoperfusion.

Figure 2 illustrates the estimated risk of FHH across the different graft weight. Risk was calculated as the moving weighted average of the FHH indicator variable (1 = FHH, 0 = no FHH), using a Gaussian kernel with a standard deviation of 300 g. The x-axis indicates the observed graft weight, with grafts without FHH shown in green and grafts with FHH in red.

### 3.3. Primary Outcome Measures

The median time from LT to CCI assessment was comparable between the FHH+ (median 34 days, IQR 23–68) and FHH− groups (median 25.5 days, IQR 18.0–39) (*p* = 0.069). Analysis of postoperative complications revealed a significantly higher mean CCI in the FHH+ group than in the FHH− group (59.8 vs. 39.3, *p* = 0.001). However, no significant association was observed between FHH grade, as classified according to the Cambridge Cradle Compression Score, and the CCI (Table 3) [2].

The multivariable linear regression of CCI had an adjusted R^2^ of 27.7% and a *p* < 0.001 for the F-test of overall significance. The distribution of the residuals and their independence from predicted values were graphically assessed. A higher MELD score (*β* = 1.07, 95% CI: 0.58–1.58, *p* < 0.001) and the presence of FHH (*β* = 13.63, 95% CI: 2.23–25.03, *p* = 0.020) remained independently associated with higher CCI (Table 4).

**Figure 3:**
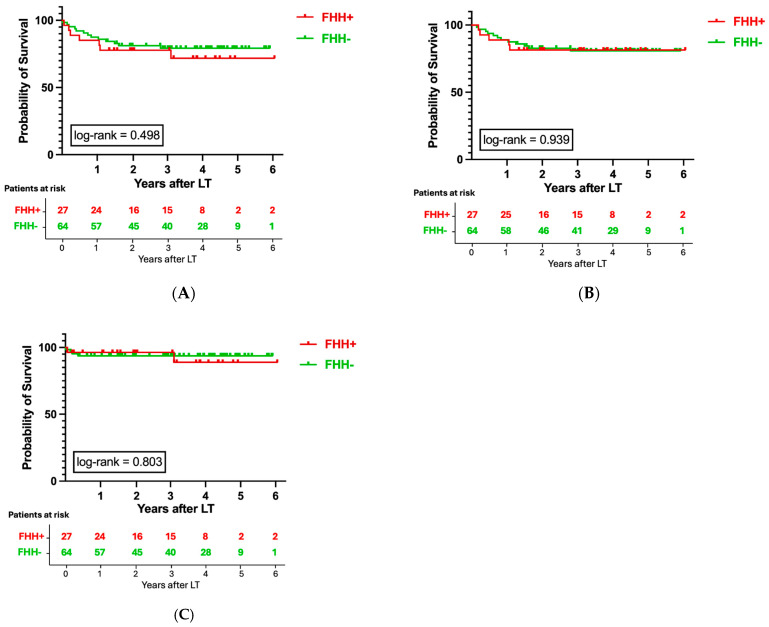
Kaplan–Meier curves comparing liver transplant recipients with (FHH+) and without FHH (FHH-). (**A**) Overall graft survival. (**B**) Overall patient surviva. (**C**) Death-censored graft survival. FHH: focal hepatic hypoperfusion.

In contrast, there were no significant differences in overall graft survival between the FHH+ and FHH- groups (Figure 3, Table 5).

### 3.4. Secondary Outcome Measures

No significant differences were observed in overall patient survival (Table 5, Figure 3), incidence of PNF, EAD, or reLT rates between the FHH+ and FHH- groups.

Although peak AST and ALT levels did not differ significantly between the groups, there was a pronounced trend toward higher median AST levels in the FHH+ group (1597 vs. 771, *p* = 0.050). In contrast, postoperative infections occurred significantly more frequently in the FHH+ group (*p* = 0.013) (Table 5) without being associated with an increased incidence of biliary complications. A detailed breakdown of the infection types is presented in Table 5.

Multivariable logistic regression analysis of postoperative infection demonstrated moderate predictive performance, with an AUC of 0.69. In the multivariable analysis, neither a high MELD score nor the presence of FHH was significantly associated with postoperative infections (Table 4).

The median length of ICU stay was significantly longer in the FHH+ group (5 vs. 3 days, *p* = 0.028). In contrast, the median overall hospital stay did not differ significantly between the groups, although there was a trend toward prolonged hospitalization in the FHH+ group (34 vs. 25.5 days, *p* = 0.069) (Table 5). The multivariable linear regression model of the log-transformed length of ICU stay demonstrated a good fit. The distribution of the residuals and their independence from predicted values were graphically assessed. The final model had an adjusted R^2^ of 20.0% and a *p* < 0.001 for the F-test of overall significance. Converted to the original scale (i.e., days), the presence of FHH increased the length of stay by 54% (95% CI: 3–131%, *p* = 0.035), and a higher MELD score increased the length of stay by 4% per point (95% CI: 2–6%, *p* < 0.001) (Table 4).

## 4. Discussion

NMP provides clear advantages over conventional static cold storage by enabling longer preservation times, greater logistical flexibility, and the opportunity for pretransplant viability assessment [4,15,23]. However, the non-physiological flow conditions inherent to NMP may result in FHH [7], which predominantly involves liver segments VII and VIII. In line with previous reports [2,24], FHH was observed in approximately 30% of grafts in our cohort.

However, the exact pathophysiology of FHH remains unclear. Current hypotheses suggest pressure-related parenchymal compression with secondary impairment of venous outflow, which consecutively reduces portal inflow to dependent liver regions during NMP. This mechanism may be particularly relevant early after perfusion initiation, when vascular resistance is increased. In this context, greater liver weight may further augment local pressure on the dependent parenchyma, thereby predisposing to the development of FHH [2,3,13,14]. Alternatively, the observed imaging pattern may reflect focal ischemic injury or infarction rather than compression alone [3].

While all previous publications consistently describe heterogeneous opacification patterns on contrast-enhanced CT scans, no standardized terminology currently exists for this phenomenon. The finding has been referred to using various terms, including ‘localized liver injury’, ‘local pressure necrosis’, ‘areas of hypoperfusion’, ‘CC’, ‘cradle effect’ and the ‘cradle sign’ [2,3,11,13,14].

In contrast to the CC definition proposed by Martin et al. [2], we included CT scans performed within 30 days after LT. Unlike Martin et al., who analyzed both DBD and donation after circulatory death (DCD) grafts [2], our cohort consisted exclusively of DBD livers, which is consistent with the legal regulations in Germany [25].

While Subramanian et al. did not observe an association between liver weight and the ‘cradle effect’, Goodnight et al. [13] and Martin et al. [2] reported an association between higher liver weight and CC. In line with these findings, a higher graft weight was identified as a significant risk factor for FHH in our cohort, demonstrating a strong association with an increased proportion of FHH (τ = 0.40, *p* < 0.001).

Notably, previously proposed liver weight thresholds varied considerably. While an abstract by Goodnight et al. reported an increased risk associated with livers weighing more than 2000 g [13], Martin et al. identified a cutoff of 1506 g for predicting CC with a sensitivity of 95% [2].

In our cohort, Youden’s J-optimized cutoff [22] for liver weight was 1958 g, yielding low sensitivity (0.48), but high specificity (0.86). Using the cutoff value of 1506 g proposed by Martin et al. [2], our analysis yielded a sensitivity of 0.81 and a specificity of 0.36, suggesting good sensitivity but limited discriminative ability due to poor specificity.

Importantly, 23% of donor livers in our cohort exceeded 1958 g and 69% exceeded 1506 g, indicating that both our Youden’s J-optimized cutoff and the threshold proposed by Martin et al. [2] may have limited applicability for routine risk stratification. Therefore, given the high proportion of heavy grafts and increasing use of high-risk donors, reliance solely on weight-based cutoffs appears insufficient for real-world clinical decision-making. Moreover, based on the current evidence, definitive conclusions regarding the use of NMP in heavier grafts cannot yet be drawn.

Due to the limited sample size, it was not possible to determine the functional relationship between graft weight and the occurrence of FHH. However, further studies are required to address this issue.

In our study, NMP duration did not contribute to the occurrence of FHH. The mean NMP duration was 14.4 ± 4.9 h, which is substantially longer than the perfusion times reported by Martin et al. (8.8 ± 2.4 h and 8.6 ± 3.5 h) [2], underscoring that our cohort represents a long-term perfusion setting.

In addition to radiological features, FHH was associated with a significantly higher incidence of postoperative infections in univariate analysis (*p* = 0.013). However, this association was not confirmed in multivariable analysis, suggesting that the observed difference may have been influenced by other clinical factors. Accordingly, our findings do not support an independent association between FHH and postoperative infection. A more detailed analysis of infection subtypes revealed that despite a significantly higher overall incidence of postoperative infections in the FHH+ group, the incidence of surgical site infections, urinary tract infections, bloodstream infections, intra-abdominal infections, infections of unknown origin, and cholangitis did not differ significantly between the groups. In contrast, pneumonia occurred significantly more frequently in patients with FHH (*p* = 0.014), likely reflecting the significantly prolonged ICU length of stay in the FHH+ group, which is associated with an increased risk of infectious complications [4,25], kidney injury [26], and delayed postoperative recovery [27] in LT recipients. Importantly, no increase in the incidence of infections caused by multidrug-resistant Gram-negative organisms was observed in either group.

FHH was associated with significantly higher CCI scores and prolonged ICU stay, indicating a greater burden of postoperative morbidity. Although we could not demonstrate an effect on survival in our cohort, elevated postoperative morbidity quantified by CCI remains prognostically relevant, as previous studies have shown that higher CCI values at discharge are associated with significantly increased 1-year graft loss and impaired long-term survival after LT [28,29,30]. While the elevated CCI observed in the FHH+ group suggests increased morbidity, no causal relationship can be inferred, and this association should be investigated in future studies.

Despite its potential impact on early postoperative outcomes, FHH was not associated with impaired graft or patient survival. This aligns with previous studies reporting a limited short-term impact, particularly in high-volume centers with optimized perioperative care [2,24]. These findings highlight the critical importance of effective failure-to-rescue prevention strategies and underscore the need for further research on targeted approaches for risk mitigation.

Importantly, our data expand the existing evidence by providing the first long-term follow-up results of up to six years, thereby offering novel insights into the sustained clinical course beyond the early postoperative period.

At our center, heavier grafts are approached with caution when considering NMP. Nevertheless, in carefully selected cases, such as the allocation of high-risk liver grafts to recipients with a low MELD score, a thorough and individualized risk–benefit assessment may justify the use of NMP, provided that the anticipated survival benefit outweighs the potential risk of FHH-related complications.

As this was a single-center retrospective study, our analysis had three main limitations: limited data (scope and size), selection bias (due to the specific population), and confounding (due to lack of randomization). Limited data reduce statistical power, which is critical for rare events, such as PNF or early reLT. Selection bias is likely to be minimal because our cohort closely resembles previously reported populations in terms of baseline characteristics, FHH prevalence, and outcomes [2]. These differences include restrictions on DBDs owing to legal requirements in Germany [25].

Another limitation stems from our inclusion criterion of a CT scan within 30 days post-transplantation. Without routine CT protocol, subclinical FHH cases may have been missed. However, routine twice-daily duplex ultrasound and additional CT imaging prompted by clinical, laboratory, or ultrasound abnormalities resulted in cross-sectional imaging in 77.2% of patients within 30 days post-transplantation, likely providing a realistic estimate of FHH incidence.

Additionally, exclusion of patients with vascular complications may have obscured associations between FHH and an increased risk of portal vein thrombosis or hepatic artery thrombosis, or vice versa, if vascular events represent risk factors for FHH.

Confounding was addressed wherever possible using multivariate analyses; however, not all relevant variables were documented consistently. Data on micro- and macro- steatosis were missing for a substantial proportion of the cohort, precluding the assessment of their impact on FHH or other outcomes. This represents an important limitation, as graft steatosis is a well-established marker of graft quality and has been associated with heightened susceptibility to ischemia–reperfusion injury, impaired microcirculatory function, altered perfusion characteristics during machine perfusion, and impaired graft performance after transplantation [31,32,33]. Consequently, the potential contribution of graft steatosis to the development of FHH could not be evaluated. Therefore, the observed association between graft weight and FHH should be interpreted with caution, as it may partly reflect differences in graft quality rather than an independent effect of graft weight itself.

In addition, the absence of standardized imaging follow-up and histopathological confirmation further limits the interpretability of the radiological findings.

Finally, the observational design of this study precludes conclusions regarding causality between FHH and postoperative morbidity.

## 5. Conclusions

In summary, FHH was not associated with impaired graft survival but was associated with higher CCI scores and prolonged ICU stays. Whether these associations reflect a causal relationship cannot be determined from the present observational study. While liver weight may contribute to the risk of FHH, reliance on weight-based thresholds is unlikely to provide adequate guidance for clinical decision making. Therefore, based on the currently available evidence, neither a specific liver weight cutoff nor the avoidance of NMP in heavier grafts can be recommended. Further prospective studies are required to elucidate the pathophysiological mechanisms underlying FHH and to better define its clinical significance.

## Figures and Tables

**Table 1:** Donor and recipient characteristics stratified for occurrence of focal hepatic hypoperfusion.

	FHH+ n = 27	FHH- n = 64	*p*-Value
Donor age [years, mean ± SD]	58.8 ± 15.1	58.4 ± 16.9	0.817 ^c^
Donor sex [% male]	59.3	46.9	0.360 ^b^
Donor BMI [kg/m^2^, median (Q0.25, Q0.75)]	28 (25, 31)	26 (23, 28.8)	0.030 ^c^
Donor resuscitation [%]	44.4	32.8	0.343 ^b^
Graft weight [g, mean ± SD]	1847 ± 471.1	1634 ± 367.6	0. 043 ^a^
ET-DRI [mean ± SD]	1.9 ± 0.4	1.8 ± 0.3u	0.824 ^a^
Allocation [n (%)]			0.131 ^b^
Standard	14 (51.9)	27 (42.2)	
REAL	3 (11.1)	20 (31.3)	
Rescue	10 (37.0)	17 (26.6)	
Recipient age [years, mean ± SD]	56.6 ± 10.8	55.5 ± 12.5	0.639 ^c^
Recipient sex [% male]	66.7	51.6	0.792 ^b^
Recipient BMI [kg/m^2^, median (Q0.25, Q0.75]	27.9 (25.6, 31.8)	26 (22.2, 29.9)	0.067 ^a^
MELD score [median (Q0.25, Q0.75)]	23 (14, 32)	18 (11.3, 26.8)	0.194 ^c^
CIT [min, mean ± SD]	433.5 ± 92.1	411.6 ± 95.7	0.310 ^a^
WIT [min, mean ± SD]	48.3 ± 19.7	44.2 ± 11.9	0.624 ^c^
NMP time [min, mean ± SD]	893 ± 302.1	851.7 ± 295	0.552 ^a^
Indication for LT [n, (%)]			
ALF	3 (11)	4 (6)	0.419 ^b^
Alcoholic cirrhosis	9 (33)	15 (23)	0.435 ^b^
Autoimmune hepatitis	1 (4)	6 (9)	0.669 ^b^
Viral hepatitis	5 (19)	8 (13)	0.517 ^b^
HCC	9 (33)	18 (28)	0.624 ^b^
Cholestatic liver disease	4 (15)	16 (25)	0.408 ^b^
Others	5 (19)	15 (23)	0.783 ^b^

Data are presented as relative frequencies, mean ± standard deviation (SD), or median with interquartile range (Q_0.25_, Q_0.75_) and compared using ^a^ Student’s *t*-test, ^b^ Fisher’s exact test and ^c^ Mann–Whitney U test. A *p*-value < 0.05 was considered statistically significant. FHH, focal hepatic hypoperfusion; FHH+, grafts with occurrence of FHH, FHH-, grafts without occurrence of FHH; BMI, body mass index; ET-DRI, Eurotransplant donor risk index, REAL, recipient-oriented extended allocation; MELD, model for end-stage liver disease; CIT, cold ischemia time; WIT, warm ischemia time, NMP, normothermic machine perfusion; LT, liver transplantation; ALF, acute liver failure; HCC, hepatocellular carcinoma.

**Table 2:** Exploratory analyses regarding possible predictors of focal hepatic hypoperfusion.

Predictor	Multivariable Logistic Regression of Focal Hepatic Hypoperfusion		Rank Correlation with Volume % of Focal Hepatic Hypoperfusion	
	OR (95%-CI)	*p*-Value	tau (95%-CI)	*p*-Value
Donor age (years)	-	−	−0.16 (−0.44–0.12)	0.260
Donor CPR (yes vs. no)	-	−	−0.13 (−0.46–0.21)	0.452
CIT (h)		−	0.00 (−0.32–0.32)	0.982
WIT (min)		−	0.33 (0.09–0.57)	0.018
NMP time (h)	-	−	0.07 (−0.24–0.38)	0.628
Graft weight (100 g)	1.14 (1.02–1.29)	0.026	0.40 (0.13–0.66)	<0.001

Empty cells indicate the variable was removed for the respective outcome during backwards elimination; OR, odds ratio; CI, confidence interval; CPR, cardiopulmonary resuscitation; CIT, cold ischemia time; WIT, warm ischemia time; NMP, normothermic machine perfusion.

**Table 3:** Grading of FHH severity according to the Cambridge Cradle Compression score and corresponding CCI values.

Grade of FHH [2]	n (%)	CCI [Mean ± SD]	*p*-Value
1	8 (30)	52.8 ± 32	0.397
2	12 (44)	58.5 ± 10.8	
3	7 (26)	70.1 ± 22.2	

Data are presented as absolute and relative frequencies or mean ± standard deviation. (SD) and compared using Analysis of Variance (ANOVA). FHH, focal hepatic hypoperfusion; CCI Comprehensive Complication Index.

**Table 4:** Multivariable linear regression analyses of Comprehensive Complication Index, log-transformed length of ICU stay and multivariable logistic regression of postoperative infection.

	CCI		Log-Transformed Length of ICU Stay		Postoperative Infection	
Predictor	Slope (95%-CI) *p*-Value		Slope (95%-CI) *p*-Value		OR (95%-CI) *p*-Value	
FHH	13.63 (2.23–25.03)	0.020	0.43 (0.03–0.84)	0.035	2.65 (0.84–10.18)	0.116
Recipient age	0.25 (−0.17–0.68)	0.233	−0.01(−0.02–0.01)	0.304	-	-
Recipient sex	-	-	-	-	-	-
Recipient BMI	-	-	-	-	-	-
MELD score	1.07 (0.58–1.58)	<0.001	0.04 (0.02–0.06)	<0.001	1.05 (1.00–1.10)	0.074
Donor CPR	-	-	−0.24 (−0.63–0.14)	0.216	-	-
Donor age	-	-	-	-	-	-
Donor sex	-	-	-	-	-	-
Donor BMI	0.73 (−0.12–1.57)	0.167	-	-	1.08(0.99–1.21)	0.120
CIT	2.79 (0.40–5.98)	0.086	0.05 (−0.06–0.17)	0.407	-	-

Empty cells indicate the variable was removed for the respective outcome during backwards elimination. CCI, comprehensive complication index; ICU, intensive care unit; OR, odds ratio; CI, confidence interval; FHH, focal hepatic hypoperfusion; BMI, body mass index; MELD, model for end stage liver disease; CPR, cardiopulmonary resuscitation; CIT, cold ischemia time.

**Table 5:** Clinical Outcome.

	FHH+ n = 27	FHH- n = 64	*p*-Value
CCI [ mean ± SD ]	59.8 ± 24.9	39.3 ± 27	0.001 ^c^
Patient survival [ % ]			0.939 ^d^
30 d	100	100	
90 d	92.6	96.9	
1 yr	88.9	89.1	
2 yrs	81.5	82.7	
3 yrs	81.5	80.8	
4 yrs	81.5	80.8	
5 yrs	81.5	80.8	
Overall graft survival [ % ]			0.498 ^d^
30 d	96.3	98.4	
90 d	88.9	95.3	
1 yr	85.2	87.5	
2 yrs	77.8	81.2	
3 yrs	77.8	79.2	
4 yrs	71.8	79.2	
5 yrs	71.8	79.2	
Death-censored graft survival [ % ]			0.803 ^d^
30 d	96.3	98.4	
90 d	96.3	95.3	
1 yr	96.3	93.8	
2 yrs	96.3	93.8	
3 yrs	96.3	93.8	
4 yrs	88.9	93.8	
5 yrs	88.9	93.8	
EAD [ n, (%) ]	11 (41)	20 (31)	0.469 ^b^
PNF [ n, (%) ]	0 (0)	2 (3)	>0.999 ^b^
reLT within 1 yr [ n, (%) ]	2 (7)	2 (3)	0.579 ^b^
Postoperative infection [ n, (%) ]	24 (89)	40 (63)	0.013 ^b^
Intra-abdominal	6 (22)	7 (11)	0.194 ^b^
Bloodstream	2 (7)	4 (6)	>0.999 ^b^
Cholangitis	14 (52)	18 (28)	0.053 ^b^
Pneumonia	7 (26)	4 (6)	0.014 ^b^
Surgical site *	0 (0)	2 (3)	>0.999 ^b^
Unknown origin	0 (0)	5 (8)	0.317 ^b^
Urinary tract	2 (7)	12 (19)	0.127 ^b^
Acute rejection [ n, (%) ]	6 (22)	11 (17)	0.569 ^b^
Peak AST POD 1–7	1597	771	0.050 ^c^
[ U/L, median (Q_0.25_, Q_0.75_) ]	(679, 2797)	(566, 1859)	
Peak ALT POD 1–7	734	565	0.071 ^c^
[ U/L, median (Q_0.25_, Q_0.75_) ]	(420, 1255)	(337, 835)	
Length of ICU stay	5	3	0.028 ^c^
[ days, median (Q_0.25_, Q_0.75_) ]	(3 , 14)	(2 , 6)	
Length of hospital stay	34	25.5	0.069 ^c^
[ days, median (Q_0.25_, Q_0.75_) ]	(23 , 68)	(18, 39)	

Data are presented as either absolute and relative frequencies, mean ± standard deviation (SD), or median with interquartile range (Q_0.25_, Q_0.75_) and compared using ^b^ Fisher’s exact test, ^c^ Mann–Whitney U test and ^d^ log rank test. A *p*-value < 0.05 was considered statistically significant. FHH, focal hepatic hypoperfusion; FHH+, grafts with occurrence of FHH; FHH-, grafts without occurrence of FHH; CCI comprehensive complication index; EAD, early allograft dysfunction; PNF, primary nonfunction; reLT, retransplantation; AST, Aspartate aminotransferase; ALT, Alanine aminotransferase; ICU, intensive care unit. * Requiring antimicrobial therapy.

## Data Availability

The data presented in this study are available on request from the corresponding author.

## Supplementary Materials

The following supporting information can be downloaded at: https://www.mdpi.com/article/10.3390/bioengineering13070729/s1, Table S1: Comparison of donor and recipient characteristics and post-transplant outcomes between patients with CT imaging within 30 days after transplantation (CT+, n = 91) and those without CT imaging (CT-, n=34).

## Abbreviations

The following abbreviations are used in this manuscript:

ALT	Alanine Aminotransferase
AST	Aspartate Aminotransferase
BMI	body mass index
BSA	body surface area
CC	cradle compression
CCI	Comprehensive Complication Index
CI	confidence interval
CIT	cold ischemia time
CPR	cardiopulmonary resuscitation
CT	computed tomography
DBD	donation after brain death
DCD	donation after circulatory death
ET-DRI	Eurotransplant Donor Risk Index
EAD	early allograft dysfunction
ENIS	Eurotransplant Network Information System
FHH	focal hepatic hypoperfusion
HR	hazard ratio
ICU	intensive care unit
LT	liver transplantation
MELD	Model for End- Stage Liver Disease
MRCP	magnetic resonance cholangiopancreatography
MRGN	multidrug-resistant Gram-negative organisms
NMP	normothermic machine perfusion
OR	odds ratio
PNF	primary non-function
POD	postoperative day
REAL	recipient-oriented extended allocation
reLT	retransplantation
SD	standard deviation
