# Supplementary material for: Focal Hepatic Hypoperfusion After Normothermic Machine Perfusion of Liver Grafts Is Associated with a Higher Comprehensive Complication Index
Source: Bioengineering (Basel). 2026 Jun 24;13(7):729. doi: 10.3390/bioengineering13070729 (PMC13405741; doi:10.3390/bioengineering13070729)
Supplement: Supplementary file 1 [file bioengineering-13-00729-s001.zip › bioengineering-4341069-supplementary.pdf]

Supplementary Material

Table S1. Comparison of donor and recipient characteristics and post-transplant outcomes between patients with CT imaging within 30 days after transplantation (CT+, n = 91) and those without CT imaging (CT-, n=34)

	**CT+ n= 91**	**CT- n= 34**	***p*-value**
**Donor age [years, mean ± SD]**	58.4 ± 16.3	59.5 ± 17.4	0.650 [c]
**Donor sex [% male]**	50.5	55.9	0.689 [b]
**Donor BMI [kg/m², median ($Q_{0.25}$, $Q_{0.75}$)]**	26 (24-31)	26 (24-32.3)	0.972 [c]
**Donor resuscitation [%]**	36.3	29	0.531 [b]
**Graft weight [g, mean ± SD]**	1697 ± 410	1759 ± 414	0.408 [c]
**ET-DRI [mean ± SD]**	1.8 ± 0.3	1.8 ± 0.3	0.776 [a]
**Allocation [n (%)]**			
Standard	41 (45)	13 (38)	0.373 [b]
REAL	23 (25)	13 (38)	
Rescue	27 (30)	8 (24)	
**Recipient age [years, mean ± SD]**	55.8 ± 12.0	55.8 ± 13.0	0.737 [c]
**Recipient sex [% male]**	56.0	79.4	**0.022** [b]
**Recipient BMI [kg/m², median (Q0.25, Q0.75)]**	26.9 (23-30.3)	26.3 (22.7-29.8)	0.746 [c]
**MELD score [median (Q0.25, Q0.75)]**	18 (12-29)	17.5 (10.5-25.3)	0.529 [c]
**CIT [min, mean ± SD]**	418.1 ± 94.7	427.4 ± 99.7	0.638 [a]
**WIT [min, mean ± SD]**	45.4 ± 14.7	43.1 ± 12.1	0.646 [c]
**NMP time [min, mean ± SD]**	864.0 ± 296.1	835.2 ± 257.5	0.596 [a]
**Indication for LT [n, (%)]**			
ALF	7 (8)	0 (0)	0.188 [b]
Alcoholic cirrhosis	24 (26)	11 (32)	0.510 [b]
Autoimmune hepatitis	7 (8)	1 (3)	0.446 [b]
Viral hepatitis	13 (14)	8 (24)	0.282 [b]
HCC	27 (30)	13 (38)	0.393 [b]
Cholestatic liver disease	20 (22)	2 (6)	**0.037** [b]
Others	20 (22)	9 (26)	0.637 [b]
**CCI [mean ± SD]**	39.3 ± 27.1	34.2 ± 27.4	0.382 [a]
**Patient survival [%]**			
30 d	100	97.1	
90 d	95.6	97.1	
1 yr	89.0	91.2	
2 yrs	82.3	88.2	0.359 [d]
3 yrs	80.8	88.2	
4 yrs	80.8	88.2	
5 yrs	80.8	88.2	
**Overall graft survival [%]**			
30 d	97.8	97.1	
90 d	93.4	97.1	
1 yr	86.8	91.2	
2 yrs	80.1	88.2	
3 yrs	78.6	88.2	
4 yrs	77.0	88.2	0.200 [d]
	77.0	88.2	

5 yrs			
**Death-censored graft survival [%]**			
30 d	97.8	100	
90 d	95.6	100	
1 yr	94.5	100	
2 yrs	94.5	97.1	
3 yrs	94.5	97.1	0.383 [d]
4 yrs	92.5	97.1	
5 yrs	92.5	97.1	
**EAD [n, (%)]**	31 (34)	10 (29)	0.674 [b]
**PNF [n, (%)]**	2 (2)	1 (3)	>0.999 [b]
**reLT within 1 yr [n, (%)]**	4 (4)	0 (0)	0.574 [b]
**Postoperative infection [n, (%)]**	64 (70)	24 (71)	>0.999 [b]
Intra-abdominal	13 (14)	2 (6)	0.352 [b]
Bloodstream	6 (7)	0 (0)	0.188 [b]
Cholangitis	32 (35)	8 (18)	0.282 [b]
Pneumonia	11 (12)	4 (12)	>0.999 [b]
Surgical site*	2 (2)	0 (0)	>0.999 [b]
Unknown origin	5 (6)	2 (6)	>0.999 [b]
Urinary tract	14 (15)	8 (18)	0.300 [b]
**Acute rejection [n, (%)]**	17 (19)	2 (6)	0.096 [b]
**Peak AST POD 1-7 [U/l, median ($Q_{0.25}$, $Q_{0.75}$)]**	895 (580.3-2155)	990 (447-1684)	0.402 [c]
**Peak ALT POD 1-7 [U/l, median ($Q_{0.25}$, $Q_{0.75}$)]**	617.5 (353.3-973)	622 (306.5-895.5)	0.799 [c]
**Length of ICU stay [days, median ($Q_{0.25}$, $Q_{0.75}$)]**	4 (2-8)	3 (2-4)	0.193 [c]
**Length of hospital stay [days, median ($Q_{0.25}$, $Q_{0.75}$)]**	29 (19-44)	18.5 (14-26)	**0.001** [c]

Data are presented as either absolute and relative frequencies, mean ± standard deviation (SD), or median with interquartile range ($Q_{0.25}$, $Q_{0.75}$) and compared using [a] Student´s *t*-test, [b] Fisher´s exact test, [c] Mann-Whitney U test and [d] log rank test. A *p*-value < 0.05 was considered statistically significant. BMI, body mass index; ET-DRI, Eurotransplant donor risk index, REAL, recipient oriented extended allocation; MELD, model for end-stage liver disease; CIT, cold ischemia time; WIT, warm ischemia time, NMP, normothermic machine perfusion; LT, liver transplantation; ALF, acute liver failure; HCC, hepatocellular carcinoma, CCI comprehensive complication index; EAD, early allograft dysfunction; PNF, primary non function; reLT, retransplantation; AST, Aspartate aminotransferase; ALT, Alanine aminotransferase; ICU, intensive care unit,

* Requiring antimicrobial therapy
